# Interaction of hnRNP K with MAP 1B-LC1 promotes TGF-β1-mediated epithelial to mesenchymal transition in lung cancer cells

**DOI:** 10.1186/s12885-019-6119-x

**Published:** 2019-09-06

**Authors:** Liping Li, Songxin Yan, Hua Zhang, Min Zhang, Guofu Huang, Miaojuan Chen

**Affiliations:** 1 Department of Clinical Laboratory, The Third Affiliated Hospital of Nanchang University, Jiangxi Nanchang, 330008 People’s Republic of China; 2 Jiangxi Province Key Laboratory of Laboratory Medicine, Department of Clinical Laboratory, Nan Chang, 330006 People’s Republic of China; 3 Medical College of Nanchang University, Jiangxi Nanchang, 330006 People’s Republic of China; 4 Guangzhou Institute of Pediatrics, Guangzhou Women and Children’s Medical Center, Guangzhou Medical University, Guangzhou, 510632 China

**Keywords:** Epithelial-to-mesenchymal transition, Heterogeneous nuclear ribonucleoprotein K, Microtubule-associated protein 1B light chain, Transforming growth factor-β 1, Non-small-cell lung cancer

## Abstract

**Backgrounds:**

Heterogeneous ribonucleoproteins (hnRNPs) are involved in the metastasis-related network. Our previous study demonstrated that hnRNP K is associated with epithelial-to-mesenchymal transition (EMT) in A549 cells. However, the precise molecular mechanism of hnRNP K involved in TGF-β1-induced EMT remains unclear. This study aimed to investigate the function and mechanism of hnRNP K interacted with microtubule-associated protein 1B light chain (MAP 1B-LC1) in TGF-β1-induced EMT.

**Methods:**

Immunohistochemistry was used to detect the expression of hnRNP K in non-small-cell lung cancer (NSCLC). GST-pull down and immunofluorescence were performed to demonstrate the association between MAP 1B-LC1 and hnRNP K. Immunofluorescence, transwell assay and western blot was used to study the function and mechanism of the interaction of MAP 1B-LC1 with hnRNP K during TGF-β1-induced EMT in A549 cells.

**Results:**

hnRNP K were highly expressed in NSCLC, and NSCLC with higher expression of hnRNP K were more frequently rated as high-grade tumors with poor outcome. MAP 1B-LC1 was identified and validated as one of the proteins interacting with hnRNP K. Knockdown of MAP 1B-LC1 repressed E-cadherin downregulation, vimentin upregulation and actin filament remodeling, decreased cell migration and invasion during TGF-β1-induced EMT in A549 cells. hnRNP K increased microtubule stability via interacting with MAP 1B-LC1 and was associated with acetylated ɑ-tubulin during EMT.

**Conclusion:**

hnRNP K can promote the EMT process of lung cancer cells induced by TGF-β1 through interacting with MAP 1B-LC1. The interaction of MAP 1B/LC1 with hnRNP K may improve our understanding on the mechanism of TGF-β1-induced EMT in lung cancer.

**Electronic supplementary material:**

The online version of this article (10.1186/s12885-019-6119-x) contains supplementary material, which is available to authorized users.

## Background

Non-small-cell lung cancer (NSCLC), as the most common type of lung cancer, remains the main cause of cancer-related death in developed countries, although important advances in the treatment of NSCLC have been achieved over the past two decades [1, 2]. Metastasis and drug resistance are the main factors contributing to the failure of treatment. Lung cancer when detected are often in a metastatic stage that metastasize by lymphatic as well as blood vessels, which usually results in the incidence of recurrence and shorten survival of the patient. Metastasis is a multifaceted process by which cancer cells disseminate from the primary site and form secondary tumors at a distant site, including local invasion, intravasation, transport, extravasation, and colonization [3–5]. Although many mechanisms and involved genes/proteins in the metastasis process have been identified, the major breakthrough is still not achieved.

Epithelial-to-mesenchymal transition (EMT) is a highly regulated and complex molecular and cellular process involved in various signaling pathways and crosstalk as well as a network of transcript factors [6, 7]. The physiopathology of the EMT process is mainly dependent upon the cellular model, the environment and the EMT stimulating factors. EMT is implicated in cancer progression through activation of proliferation pathway, loss of response to apoptotic signals, gain of stem cell properties, matrix remodeling and mobility [8–10]. EMT plays a critical role in promoting metastasis in lung cancer [11]. Because of its link with metastasis and resistance to treatment, EMT has been considered as a useful prognosis and predictive marker but there is yet no clinical application in NSCLC. Thus, enhancing our knowledge of the mechanism of EMT may enable us to forward EMT charcterization to the clinics.

The heterogeneous nuclear ribonucleoprotein K (hnRNP K), as a member of hnRNP family, was first discovered using two dimensional gel of the immunopurified complex. hnRNP K serves as a docking platform for the assembly of multimolecular signaling complexes, integrating transduction pathways to nucleic acid-directed processes. Aberrant expression of hnRNP K is a common to all tumors studied. Its aberrant cytoplasmic localization is associated with a worse prognosis for patients, and its cytoplasmic accumulation strongly promotes tumor metastasis, which suggest that it is involved in cancer development and progression [12–14]. Our previous study demonstrated that hnRNPs are positive regulation nodes in the migration-related network and the connection of hnRNP K in A549 cells with EMT [15]. Recent studies revealed that long non-coding RNA interacts with hnRNP K to promote tumor metastasis [16]. However, the molecular mechanisms by which hnRNP K modulates TGF-β1-mediated EMT in lung cancer cell remain largely unclear.

In this study, to elucidate the role of hnRNP K in these intracellular processes, we used co-immunoprecipitation (Co-IP) in tandem with LCMS/MS analysis to identify the new interacting partners of hnRNP K in A549 cells during TGF-β-induced EMT. Among the identified candidates, microtubule-associated protein 1B light chain (MAP 1B-LC1/LC1) attracted our attention. LC1 was characterized as a subunit of MAP 1B was found to bind to microtubules in vivo and in vitro and induce rapid polymerization of tubulin [17, 18]. The interaction of MAP 1B/LC1 with hnRNP K may provide new insights into the molecular mechanism underlying the involvement of hnRNP K during TGF-β-induced EMT in A549 cells.

## Methods

### Antibodies

The following primary antibodies were used: monoclonal anti-GAPDH (Catalog G8795) from Sigma; monoclonal anti-LC1 (Catalog sc-136,472) and monoclonal anti-hnRNP K (Catalog sc-28,380) from Santa Cruz Biotechnology Inc.; monoclonal anti-snail (Catalog #3895S), polyclonal anti-vimentin (Catalog #5741) and polyclonal anti-E-cadherin (Catalog #3195) from Cell Signaling Technology; monoclonal anti-HSP70 (Catalog #66183–1-lg), monoclonal anti-acetylated tubulin (Catalog #66200–1-Ig) and polyclonal anti-ɑ-tubulin (Catalog #11224–1-AP) from proteintech. Goat anti-mouse Alexa Fluor 594 (Catalog #R37117) was purchased from Molecular Probes, and peroxidase-coupled secondary antibody was from Life technology.

### Plasmids

Standard PCR procedures were used to insert restriction sited into plasmids for cloning. LC1 cDNA was inserted into pGEX-6P-1. hnRNPK cDNA inserted into lentivirus packing expression vector GV341 (named as pGV341-hnRNP K) was purchased from Shanghai GenePharma (GenePharma, Shanghai, China).

### Cell cultures, transient transfection and generation of stable cell line

Human NSCLC cell lines, A549, was obtained from the American Type Culture Collection (ATCC, Manassas, VA), and cultured in F-12 K medium with 10% fetal bovine serum (FBS) at 37 °C, 5% CO_2_ in air.

For RNA interference, RNAs (siRNA) for MAP 1B-LC1 or nontargeting siRNAs were transfected using RNAiMAX (Invitrogen). The cells were then allowed to grow for another 48 h for the following experiment. MAP 1B-LC1 siRNA was obtained from Shanghai GenePharma (GenePharma, Shanghai, China). MAP 1B-LC1 siRNA1 (sense, 5′-CCACAGCAAUAGUAAGAAUTT-3′; antisense, 5′-AUUCUUACUAUUGCUGUGGTT-3′), siRNA2 (sense, 5′-GACGCUUUGUUGGAAGGAATT-3′; antisense, 5′-UUCCUUCCAACAAAGCGUCTT-3′) were chosen as the main siRNA for sufficient knockdown. All siRNA were dissolved to a final concentration of 20 μM and stored at − 20 °C.

For overexpression experiment, A549 cells were transfected with appropriate plasmids at the final concentration of 1.5 μg/mL using LTX. For experiments where cells were subjected both to RNA interference and overexpression treatments, cells were co-transfected with siRNA and plasmids.

To generate hnRNP K-overexpressing A549 cell line, cells were infected with lentivirus carrying the hnRNP K gene. After 48 h of incubation, cells were passaged three times with 10% FBS-F-12 K containing puromycin. The positively screened cell line was determined by western blot.

### SDS-PAGE and Western blotting

Protein samples were denatured at 100 °C for 5 min, separated on 10% or 12% SDS-PAGE gels at 100 V for 3 h. Then the gel was stained with a silver staining method or electro-transferred onto polyvinylidene-difluoride (PVDF) membrane (0.45 μm, Millipore). The membranes were blocked with 5% non-fat milk solution for 1 h at room temperature (RT) and incubated with primary antibody dissolved in block solution at 4 °C overnight. After washing, the membranes were incubated with horseradish peroxidase-conjugated secondary antibody corresponding to the primary antibody for 1 h at RT. Protein bands were detected by the enhanced chemiluminescence method (ECL, Millipore).

### Silver staining, in-gel digestion, LC-MS/MS and data analysis

After SDS-PAGE, proteins were detected by a silver nitrate staining protocol adapted from Wang et al. After silver staining, the protein bands were excised for tryptic in-gel digestion. Peptides were analyzed using LTQ-Orbitrap mass spectrometer operated in data-dependent mode to automatically switch between full-scan MS and MS/MS acquisition. Raw data from LC-MS/MS were automatically processed by MaxQuant 1.1.1.2 software against a IPI human protein database (V3.49) with the default setting.

### Immunoprecipitation assay

Cells were lysed in a buffer containing 20 mM Tris-HCl pH 7.5, 150 mM NaCl, 1% Triton X-100, 1 mM Na_3_VO_4_, 1 mM PMSF, and protease and phosphatase inhibitor for 30 min at 4 °C. Lysates were clarified by centrifugation at 13200 rpm for 30 min at 4 °C. Then cell lysates were precleared using 20 μL Protein A/G Plus-agarose for 30 min at 4 °C. After pre-clearing, cell lysates (1 mg) was incubated with 2 μg hnRNP K or isotype matched IgG Abs at 4 °C overnight. 30 μL of Protein A/G Plus-agarose were then added to the supernatants and incubated for 4 h at 4 °C. The immunoprecipitates and lysates were subjected to western blot using the antibody indicated.

### Recombinant proteins and GST pull-down assay

For the GST pull-down assay, the GST fusion protein was induced for 8 h in 500 mL of *E. coli* Rosseta cells by addition of 200 μM isopropyl β-D-thiogalactopyranoside (IPTG). After centrifugation, the bacterial pellet was resuspended in 50 mM Tris-HCl,150 mM NaCl, 1% Triton X-100, 2 mM EDTA and 1% lysozyme, and then ultrasonicated in ice for 10 min until the supernatants were clear. After centrifugation, the supernatant fraction was bound to 500 μL of pre-washed glutathione-Sepharose beads (GE Healthcare) for 2 h at 4 °C. The beads were washed with lysis buffer, the purity of the bound GST fusion protein was analyzed by SDS-PAGE, and its concentration was determined for the following experiment. A549 cell lysate (1 mg) was added to 50 μg of GST-MAP 1B-LC1 or GST beads in 1 mL of bacterial lysis buffer for 12 h at 4 °C. Beads were then washed four times with bacterial lysis buffer, resuspended in SDS loading buffer, and analyzed by SDS electrophoresis and western blot with anti-hnRNP K antibody.

### Immunofluorescence assay

After transfection and treatment with TGF-β1, cells grown on glass coverlips were fixed with pre-cooled methanol for 2 min at room temperature. After washing with PBS containing 2 mg/ml glycine, the cells were permeabilized with 0.1% Trintion X-100 for 10 min at RT, blocked with 10% goat normal serum for 1 h, and then incubated with the primary antibodies overnight at 4 °C. After washing with PBS containing 0.05% Tween-20 and 1% BSA, cells were incubated with the indicated secondary antibodies. Microtubulin was stained using Tubulin-Tracker. Images of cells were aquired using confocal microscope and prepared with ImageJ software.

### Migration and invasion assay

Migration and invasion assays were performed using transwell chambers with or without a Matrigel (8 μm pore size, BD, Falcon). After transfection, 3 × 10^5^ A549 cells were seeds in the upper chamber in 300 μL F-12 K medium and allowed to migrate for 3–6 h or invade for 24 h at 37 °C. F-12 K with 10% FBS was used as a chemoattractant in the lower chamber. Cells were fixed in 4% paraformaldehyde, stained with 0.1% crystal violet, and imaged (5 fields/well) using a microscope. For the quantitation of migrated or invaded cells, 5 fields of migrated cells in each well were counted.

### NSCLC patient samples and immunohistochemistry

This research was approved by the Human Ethics Committee and the Research Ethics Committee of the Third Affiliated Hospital of Nanchang University. Patients were informed that the resected specimens were stored by the hospital and potentially used for scientific research.

Total 94 tissue samples were used for this study, including 94 NSCLC and 86 adjacent non-tumor tissues. All tissues were collected from Shanghai Outdo Biotech Co. Ltd. (Outdo Biotech). All tissues were fixed in 10% buffered formalin and embedded in paraffin blocks. The pathological parameters, including gender, age, tumor size, clinical stage, differentiation, nodal metastasis and survival data, were carefully reviewed in all 94 NSCLC cases.

IHC analysis was performed using the DAKO LSAB kit (DAKO A/S, Glostrup, Denmark). Briefly, to eliminate endogenous peroxidase activity, tissue sections were deparaffinized, rehydrated and incubated with 3% H2O2 in methanol for 15 min at RT. The antigen was retrieved at 95 °C for 20 min by placing the slides in 10 mM sodium citrate buffer (pH 6.0). The slides were then incubated with hnRNP K antibody at 4 °C overnight. After incubation with secondary antibody at RT for 30 min, IHC staining was developed using 3,3′-diaminobenzidine, and Mayer’s hematoxylin was used for counterstaining. In addition, the positive tissue sections were processed with omitting of the primary antibody as negative controls.

All specimens were examined by two investigators who did not possess knowledge of the clinical data. The staining intensity of the IHC staining for hnRNP K was assessed on a scale of weak (1), medium (2) or strong (3). The staining intensity of the IHC staining for hnRNP K was assessed using histochemistry score (H-SCORE). H-SCORE is used for semiquantitative analysis for stained tissue.(H-SCORE = ∑(PI×I) = (percentage of cells of weak intensity × 1) + (percentage of cells of moderate intensity × 2) + percentage of cells of strong intensity × 3), PI shows percentage of cells of all positive cell numbers, I represents stain intensity [19]. The sample was classed as low (score < 60) or high (score > 60) hnRNP K expression.

### Statistics

Data were expressed as mean ± SD of 3 independent experiments. Differences between groups were assessed using Student’s t-test. Associations between gene expression and clinical pathologic characteristics were assessed with chi-square tests. Cumulative survival time was calculated using the Kaplan-Meier method and analysed by the log-rank test. *P* < 0.05 in all cases was considered statistically significant. All data were analyzed with the Statistical Package for the Social Science (SPSS, Chicago, IL), Version 13.0.

## Results

### Expression of hnRNP K in NSCLC tissues with different clinical and pathological characteristics

**Fig. 1:**
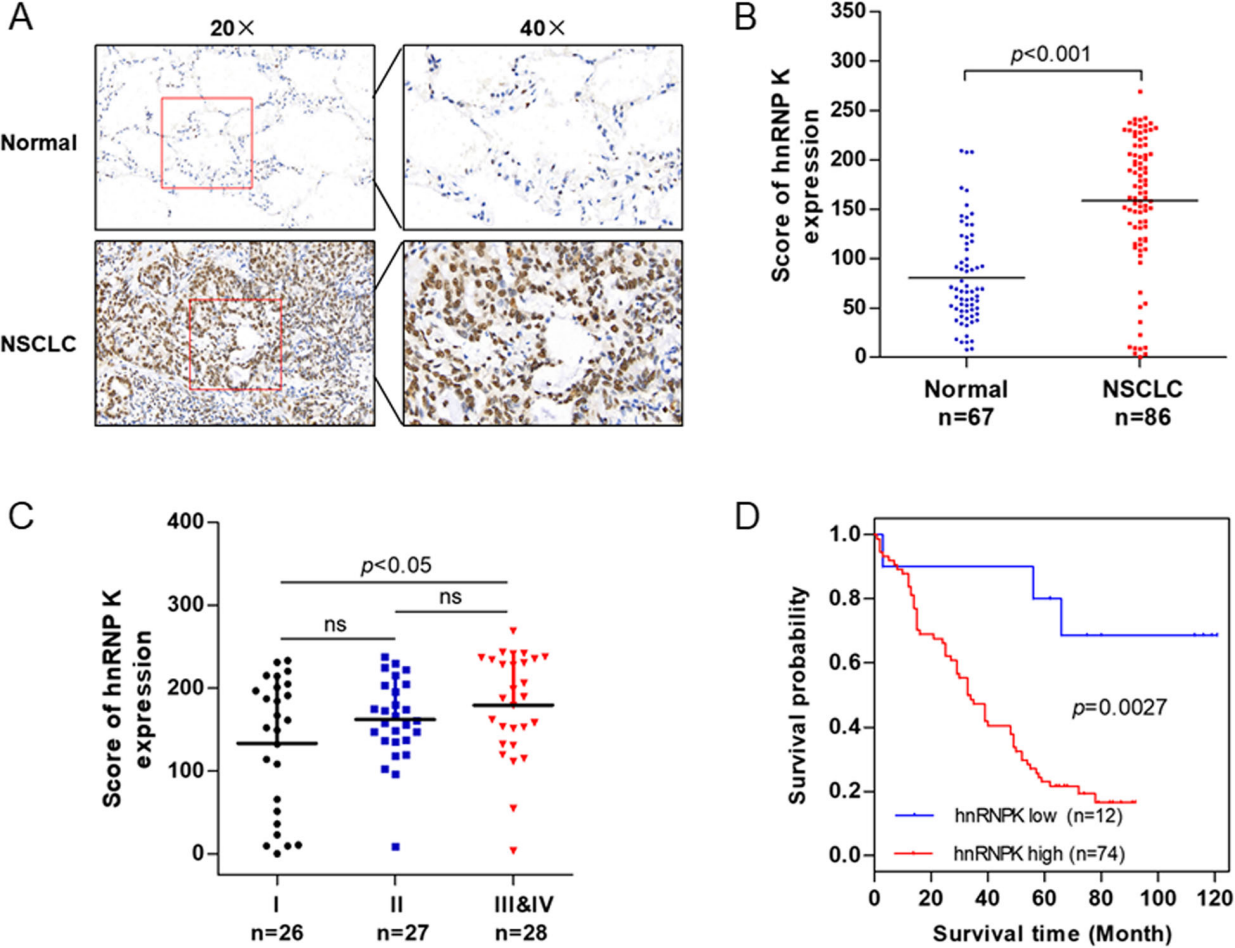
Expression levels of hnRNP K in non-small-cell lung cancer tissues and adjacent non-tumor tissues. **a** Representative images of hnRNP K immunohistochemical staining in NSCLC and adjacent non-tumor tissues. **b** IHC expression of hnRNP K quantified by expression score (0–300) in NSCLC and adjacent non-tumor tissues. *P* < 0.001. **c** The differences of IHC expression of hnRNP K quantified by expression score (0–300) in NSCLC subtype. *P* < 0.05. **d** The overall survival rates of the 86 patients with NSCLC were compared according to low- and high-hnRNPK status. Statistical significance was determined using the log-rank test

To study the expression of hnRNP K in NSCLC, sample from 94 NSCLC and 86 adjacent normal tissues were collected and detected by immunohistochemistry, and then each immunostained section was assessed using a score method. We found that hnRNP K was located predominantly in the nucleus, and the average staining and score of hnRNP K expression in NSCLC were significantly higher than those in normal tissues (Fig. 1a and b). hnRNP K expression was obviously higher in tumor stage III and IV as compared with stage I (Fig. 1c). Further we evaluated the association between hnRNP K expression and clinicopathological factors. As shown in Table 1, hnRNP K expression was significantly positively associated with tumor size, clinical stage, and tumor stage. Moreover, Kaplan-Meier survival analysis showed that overall survival significantly reduced in patients with NSCLC with increased hnRNP K expression as compared with those in patients with low hnRNP K immunostaining (Fig. 1d). These results suggested that hnRNP K was a potential prognostic marker in NSCLC.

**Table 1:** Correlation between hnRNPK expression and clinicopathologic characteristics of lung cancer patients

		hnRNPK expression		Chi-square test	
Characteristics	n	Low or none No. cases (%)	High No. cases (%)	*p* value	χ^2^
Gender					
Male	51	13	38	0.592	0.431
Female	43	11	32		
Age					
≤ 55	22	10	12	0.756	0.097
> 55	72	15	57		
Clinical Stage				0.000*	27.180
I + II	59	16	43		
III + IV	35	9	26		
T classification				0.000*	50.255
T1 + T2	71	19	52		
T3 + T4	23	5	18		
M classification					
M0	92	22	70	0.000*	1.000
M1	2	0	2		
N classification				0.000*	32.316
N0	39	11	28		
N1	55	13	42		
Vital status (at follow-up)				1.000	0.442
Alive	24	7	17		
Death	70	17	53		
Tumor size (cm)				0.000*	78.340
≤ 5	82	21	61		
> 5	12	4	8		

### Suppressing hnRNP K expression during EMT decreased cell migration and invasion

**Fig. 2:**
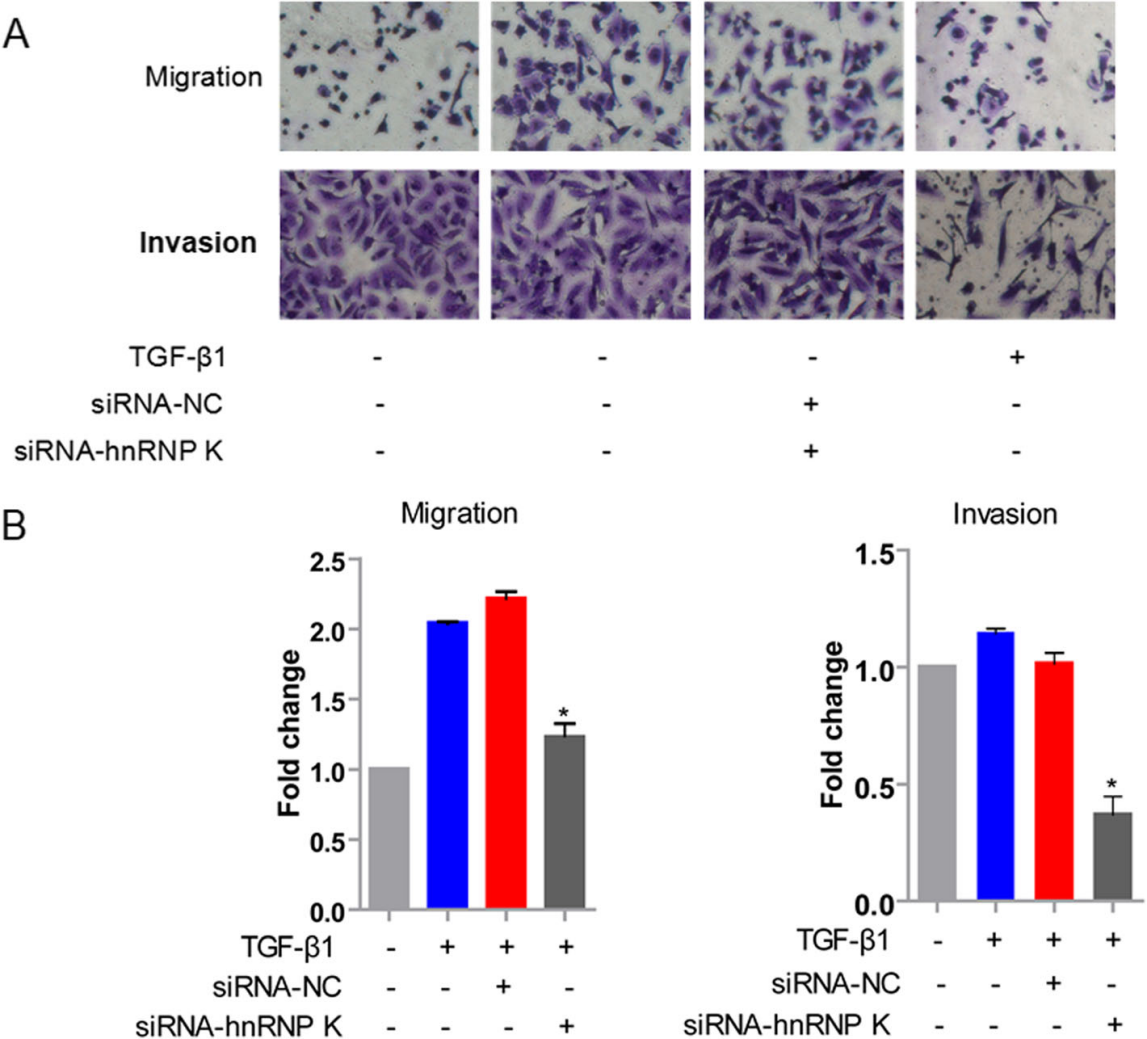
Suppression of hnRNP K expression during EMT decreased cell migration and invasion. **a** Representative images of cell migration and invasion. The cells transfected with NC siRNA or hnRNP K siRNA were seeded in the inserts with or without BD Matrigel and incubated for 3 h or 24 h. **b** The histograms showed the fold change of cell migration and invasion. Mean ± standard deviation (error bars) of three separate experiments performed in triplicate. ^*^P < 0.05 compared with cells transfected with hnRNP K siRNA

In vitro, our previous study demonstrated the association between hnRNP K and EMT. The acquisition of migratory and invasive properties is one of the phenotypic changes during EMT. To further determine the role of hnRNP K in cell migratory and invasive abilities during EMT, we observed cell migration and invasion after TGF-β1 treatment by overexpression or knockdown of hnRNP K. As shown in Fig. 2, hnRNP K overexpression or knockdown promoted or inhibited migration and invasion of A549 cells. The results demonstrated that hnRNP K was involved in regulating cell migration and invasion after TGF-β1 treatment, further confirming hnRNP K was required for TGF-β1-induced EMT in A549 cells.

### Identification of the proteins interacting with hnRNP K

**Fig. 3:**
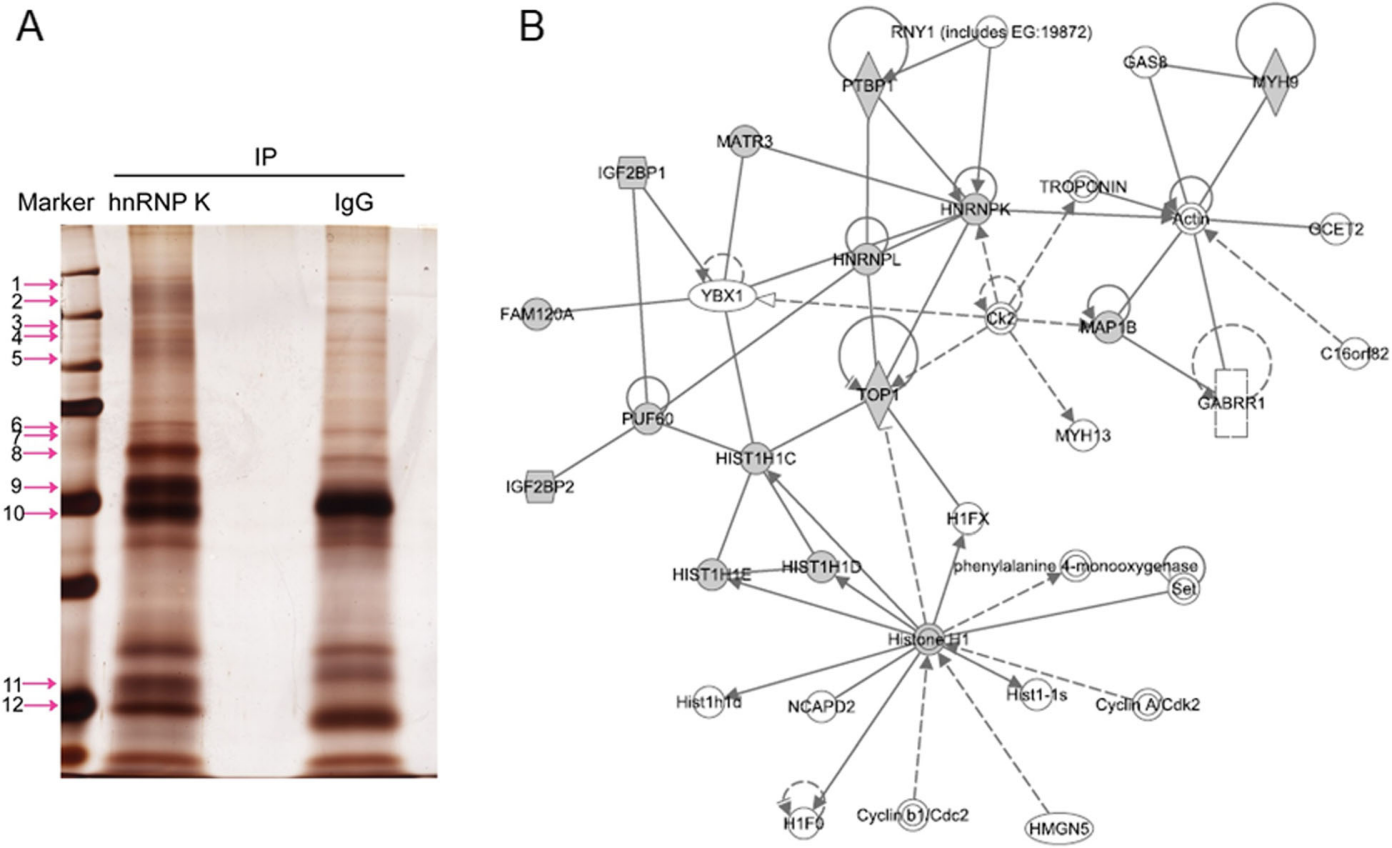
Immunoprecipitation assay with sliver staining. **a** Co-immunoprecipitation experiments were performed using A549 cell lysates with anti-hnRNP K antibody, or with non-immune IgG as negative control. The proteins were resolved on SDS-PAGE, and stained with silver staining. Arrows: the extracted bands. **b** Analysis of protein-protein interaction network of hnRNP K

To investigate the molecular mechanisms of action of hnRNP K in the cell, we used Co-IP combined with LC-MS/MC analysis to identify the new interacting partners of hnRNP K. The proteins associated with hnRNP K antibody or control IgG were separated by SDS-PAGE and stained with sliver staining (Fig. 3). Some individual bands of hnRNP K lane were extracted for in-gel digestion, with the corresponding bands of control IgG lane and then analyzed by LC-MS/MS. 22 proteins were identified as the new interacting partners of hnRNP K (Additional file 1: Table S1). These proteins included proteins related to RNA transcription and modification, RNA binding proteins, mRNP forming proteins, and cytoskeletal binding proteins. Among the identified candidates, MAP 1B-LC1/LC1 attracted our attention.

### hnRNPK interacted with MAP 1B-LC1

**Fig. 4:**
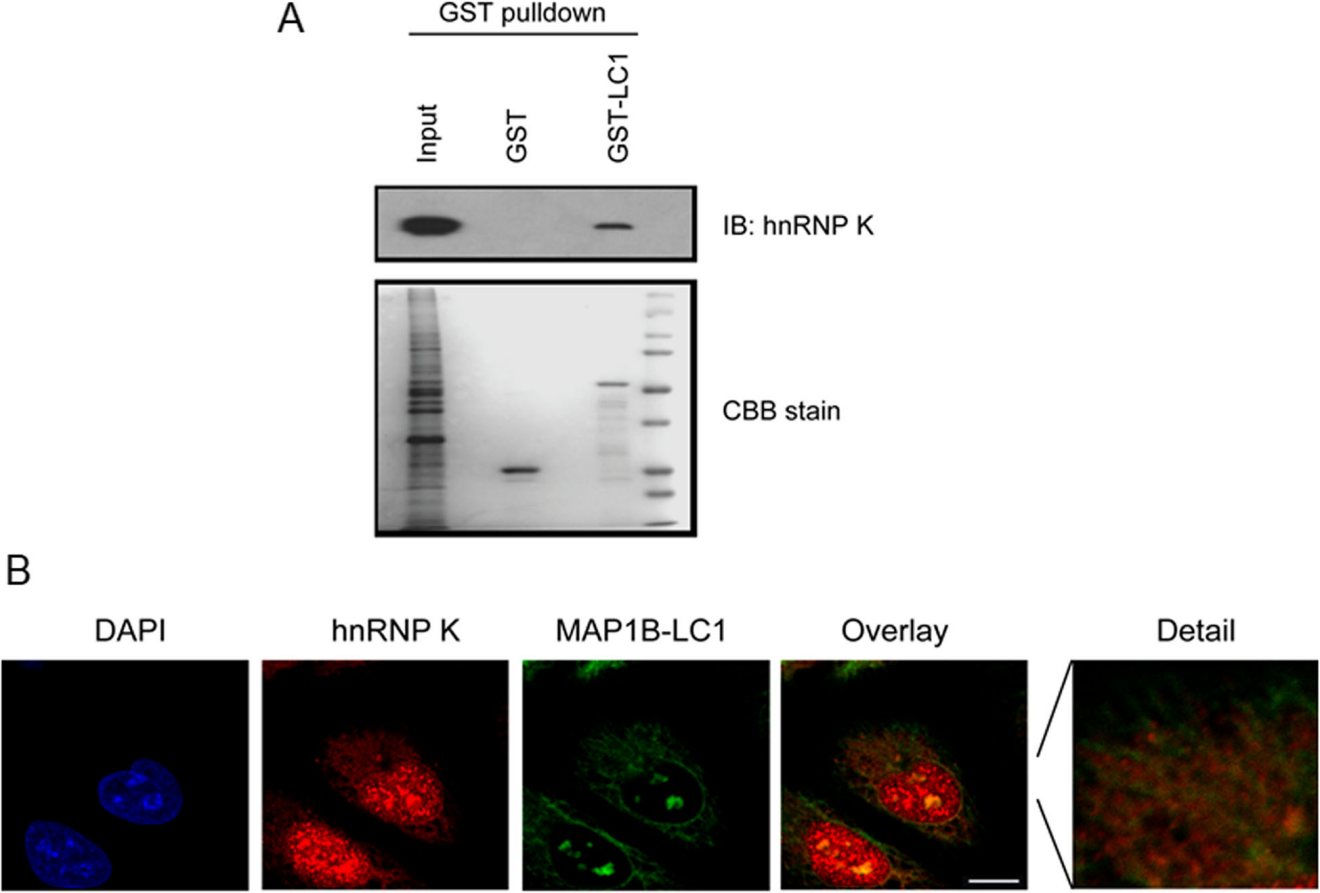
hnRNP K interacted with MAP 1B-LC1. **a** Purified GST (lane 2) or GST-MAP 1B-LC1 recombinant proteins (lane 3) were immobilized on Sepharose-Glutathione beads and incubated with A549 cell lysates. Lane 1 represents the whole cell lysates. The amounts of GST and GST-MAP 1B-LC1 used in the assays were checked by coomassie blue stainng (lower panel). **b** Co-localization of hnRNP K and MAP 1B-LC1 in A549 cells. Bar, 10 μm

To define the biochemical mechanisms that mediate hnRNP K’s action, we tested whether hnRNP K interacted with MAP 1B-LC1. For this purpose, the interaction was confirmed by GST pull-down experiment using GST-MAP 1B-LC1. The result showed that hnRNP K could be pulled down by the GST-MAP 1B-LC1 but not the GST (Fig.4a). Then, the subcelluar distribution of hnRNP K and MAP 1B-LC1 was investigated using confocal microscope. As shown in Fig. 4b, the co-localization of hnRNP K and MAP 1B-LC1 was in the microtubulin-like structure of A549 cells. These results confirmed that hnRNP K interacted with MAP 1B-LC1.

### Knockdown of MAP 1B-LC1 repressed TGF-β1-induced EMT in A549 cells

**Fig. 5:**
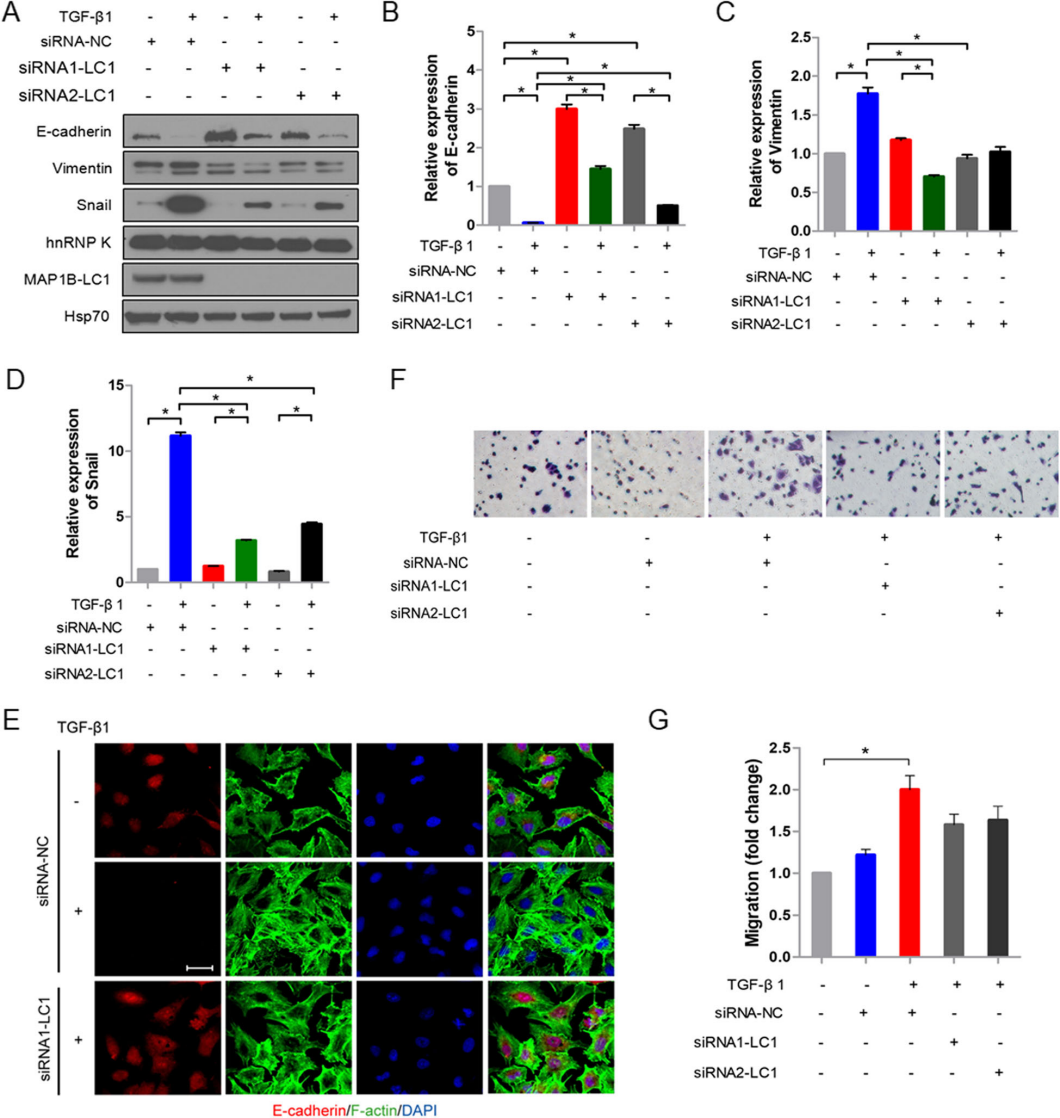
Knockdown of MAP 1B-LC1 repressed TGF-β1-induced EMT in A549 cells. **a** Western blotting analysis of MAP 1B-LC1 involved in EMT. A549 cells were transfected with MAP 1B-LC1 siRNA or NC siRNA, and stimulated with 5 ng/mL TGF-β1. **b**-**d** Quantifications of the expression of E-cadherin, vimentin and snail in A549 cells transfected with MAP 1B-LC1 siRNA or NC siRNA after TGF-β1 treatment. Mean ± standard deviation (error bars) of three separate experiments performed in triplicate. ^*^P < 0.05. **e** Immunofluorescent analysis for EMT markers E-cadherin and F-actin polarization. E-cadherin (red), F-actin (green), and DAPI (blue) staining were shown, respectively. Bar, 100 μm. **f** Representative images of cell migration. The cells transfected with NC siRNA or hnRNP K siRNA were treated with TGF-β1, seeded in the inserts and incubated for 3 h. **g** The histograms showed the fold change of cell migration. Mean ± standard deviation (error bars) of three separate experiments performed in triplicate. ^*^P < 0.05

As shown in the above results, MAP 1B-LC1 could interact with hnRNP K. Because no studies showed that MAP 1B-LC1 is involved in TGF-β1-induced EMT, we hypothesize that MAP 1B-LC1 as a microtubule-associated protein, may function as a modulator of cell migration and thus affects the EMT phenotype of A549 cells. To test this hypothesis, the EMT phenotype of A549 cells were induced by TGF-β1 after knockdown of MAP 1B-LC1 expression by RNAi. Western blotting results showed that cells transfected with MAP 1B-LC1 siRNA exhibited increased expression of epithelial marker (E-cadherin) and decreased expression of mesenchymal marker (vimentin) and transcription factor (snail) in contrast to negative control siRNA cells, verifying that knockdown of MAP 1B-LC1 repressed the EMT phenotype of A549 cells (Fig. 5a-d). The immunocytochemistry results further confirmed that the expression level of E-cadherin was downregulated in A549 cells after the treatment of TGF-β1. However, the expression of E-cadherin was restored by knocking down MAP 1B-LC1 (Fig. 5e). F-actin polarization is known as another EMT phenotype, which is related to cell migration and cytoskeleton assembly. As shown in Fig. 5e, F-actin polarization was observed in A549 cells upon TGF-β1 stimulation, whereas this polarized F-actin distribution was significantly reduced when knocking down MAP 1B-LC1. Further, our results demonstrated that TGF-β1 stimulated significantly the migrating ability of A549 cells, whereas knocking down MAP 1B-LC1 greatly affected this capacity, indicating that MAP 1B-LC1 was involved in this cellular process (Fig. 5f and g). Taken together, these results suggested that MAP 1B-LC1 was involved in TGF-β1-induced EMT in A549 cells.

### Knockdown of MAP 1B-LC1 inhibits hnRNP K-mediated EMT induced by TGF-β1

**Fig. 6:**
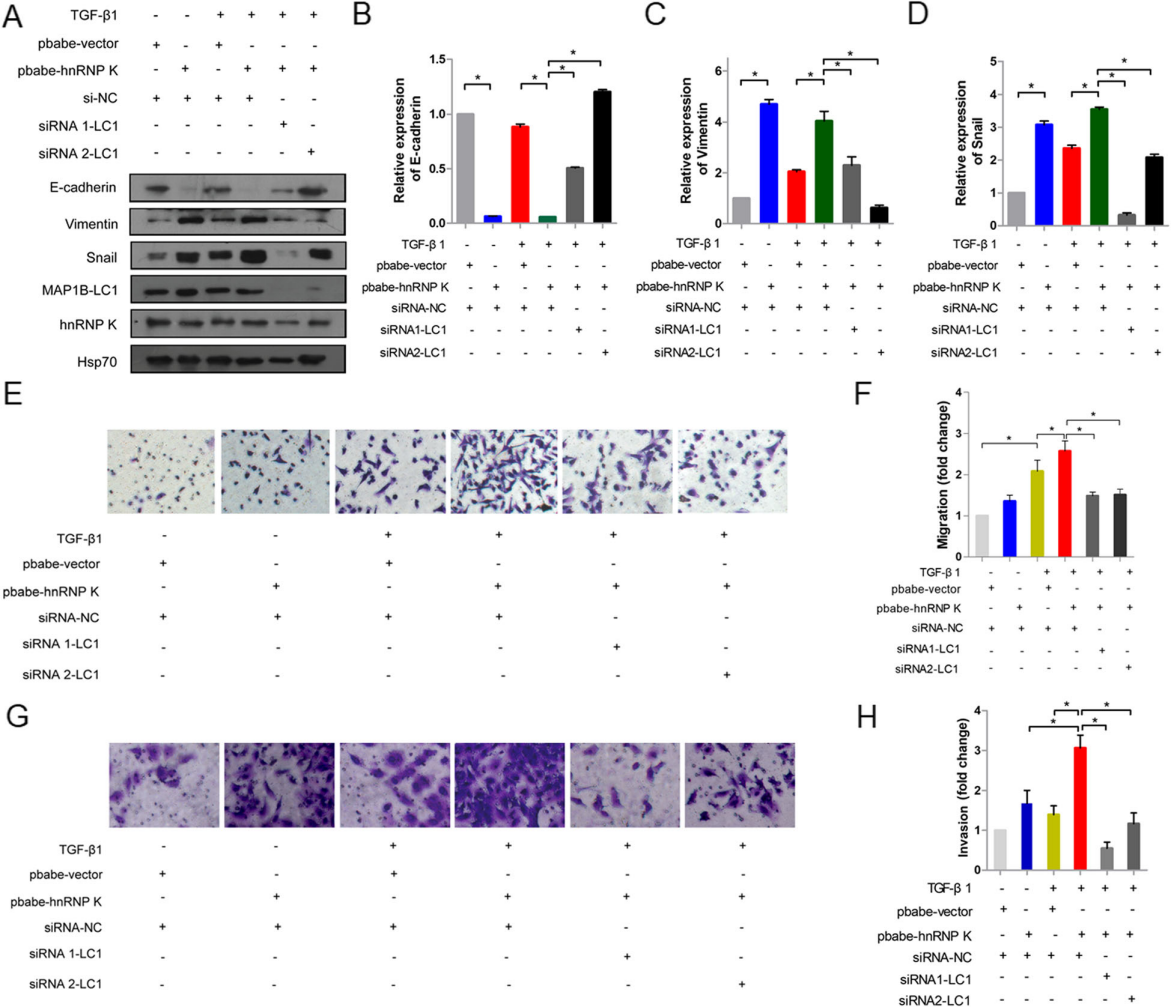
MAP 1B-LC1 was required for hnRNP K-mediated EMT induced by TGF-β1. **a** Western blotting analysis of the expression of EMT markers E-cadherin, vimentin and snail. A549 cells stably overexpressing hnRNP K or vector were transfected with NC siRNA or hnRNP K siRNA, and then stimulated with 5 ng/mL TGF-β1. **b**-**d** Quantifications of the expression of E-cadherin, vimentin and snail. Mean ± standard deviation (error bars) of three separate experiments performed in triplicate. ^*^P < 0.05. **e**, **g** Representative images of cell migration and invasion. The cells stably overexpressing hnRNP K or vector were transfected with NC siRNA or hnRNP K siRNA, seeded in the inserts with or without BD Matrigel and incubated for 3 h or 24 h. **f**, **h** The histograms showed the fold change of cell migration and invasion. Mean ± standard deviation (error bars) of three separate experiments performed in triplicate. ^*^P < 0.05

Next if the functions of hnRNP K and MAP 1B-LC1 during TGF-β1-induced EMT are linked or independent from each other, was investigated. MAP 1B-LC1 was transiently knocked down in hnRNP K-overexpressing A549 cells using siRNA and the EMT phenotype and cell migration were examined using western blot and transwell assays. As shown in Fig. 6, hnRNP K overexpression in A549 cells affected their EMT phenotype and promoted cell migration. When MAP 1B-LC1 expression was knocked down in the cells stably overexpressing hnRNP K, the expression of E-cadherin was restored, whereas vimentin and snail decreased to a level comparable to those of cells only stably overexpressing hnRNP K, and the cell migrating ability dropped down. In the other word, knockdown of MAP 1B-LC1 expression cancelled the stimulatory effect of hnRNP K overexpression, which supported our hypothesis in which these two regulatory effects were linked in the regulation of EMT induced by TGF-β1.

### hnRNP K increased microtubule stability through MAP 1B-LC1 and was associated with the acetylation of ɑ-tubulin

**Fig. 7:**
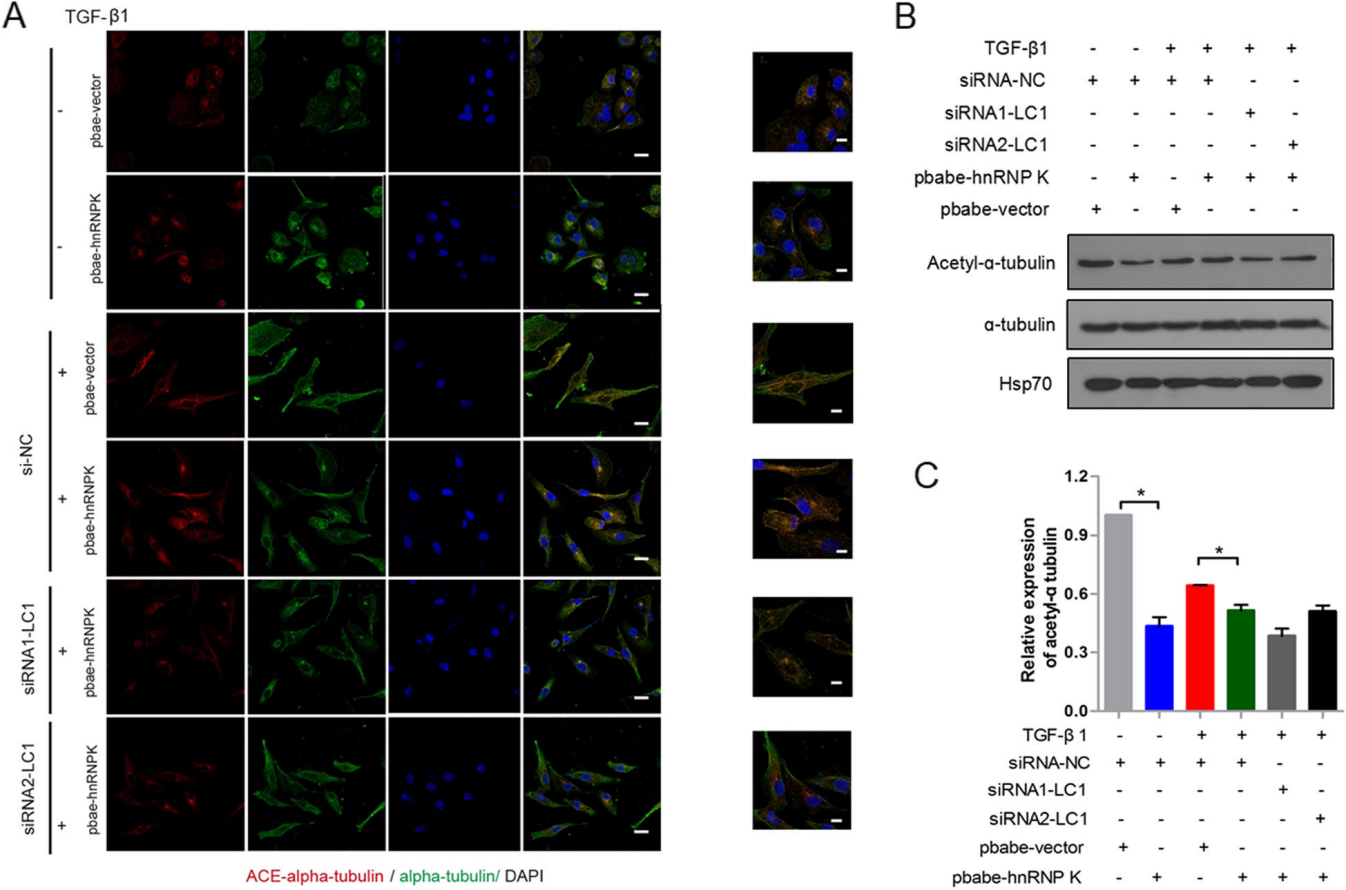
hnRNP K increased stress fibers of microtubule through MAP 1B-LC1 and was associated with the acetylation of ɑ-tubulin. **a** Immunofluorescent analysis for the formation of stress fibers. hnRNP K (red), tubulin (green), and DAPI (blue) staining were shown, respectively. **b** Western blotting analysis of the expression of acetylated-ɑ-tubulin. A549 cells stably overexpressing hnRNP K or vector were transfected with NC siRNA or hnRNP K siRNA, and then stimulated with 5 ng/mL TGF-β1. **c** Quantifications of the expression of acetylated-ɑ-tubulin. Mean ± standard deviation (error bars) of three separate experiments performed in triplicate. ^*^P < 0.05

Previous study showed that MAP 1B-LC1 has microtubule stabilizing activity [18]. These results suggested that interaction of hnRNP K with MAP 1B-LC1 may be involved in microtubule stabilization. To investigate if hnRNP K plays a role in microtubule stabilization through MAP 1B-LC1, we examined micortubule bundling in hnRNP K overexpressing A549 cells with or without knockdown of MAP 1B-LC1 by immunofluorescence. As reported in previous study, in the absence of TGF-β1 treatment, microtubules are shown as sparse, randomly oriented filaments in control cells. Upon TGF-β1 treatment, microtubules form stress fibers. Compared with control cells, hnRNP K overexpressing A549 cells display robust tubulin with bundling or increased density of microtubules radiating from the perinuclear region with or without TGF-β1 treatment. But, knockdown of MAP 1B-LC1 with siRNA could destroy microtubulin from the perinuclear region induced by overexpression of hnRNP K (Fig. 7a). These results suggested that hnRNP K promoted microtubule stabilization through MAP 1B-LC1. Acetylation of ɑ-tubulin is associated with microtubule stability and a novel regulator and marker of EMT [20, 21]. To investigate the role of hnRNP K and MAP 1B-LC1 in the acetylation of ɑ-tubulin during TGF-β1-induced EMT, we compared the levels of acetylated ɑ-tubulin in A549 cells after different treatments. The results showed that hnRNP K overexpression decreased the acetylation of ɑ-tubulin. However, when MAP 1B-LC1 expression was knocked down in the cells stably overexpressing hnRNP K, the acetylation of ɑ-tubulin was not restored (Fig. 7b and c). The result implied that hnRNP K was involved in regulating the acetylation of ɑ-tubulin mediated by other pathways but not by the binding of MAP 1B-LC1.

## Discussion

Increased hnRNPK expression is associated with malignant tumor and its aberrant cytoplasmic expression is associated with metastasis in several tumors. In this study, we showed that increased expression of hnRNP K in NSCLC was positively correlated with advanced tumor stage, and was associated with poor prognosis and served as an independent predictor of overall survival in NSCLC. Consistent with our findings, previous studiess found that high-hnRNP K expression in tumors was closely associated with poor prognosis [13, 22]. Taken together, high-hnRNP K expression levels may serve as a novel prognostic marker for advanced NSCLS.

hnRNPK works as one of mRNA translation regulators via their 3’UTR, which could alter the stability, translational activity, and subcellular localization of mRNAs [23–26]. A variety of post-translational modifications of hnRNP K exist in cytoplasm to play a regulatory role for metastasis, including phosphorylation, ubiquitination, sumoylation and methylation. hnRNP K is composed of the nuclear localization signal domain and the nuclear shuttling domain, which is modified and regulated by Erk/Src/PKC signaling pathway [27]. hnRNP K not only participates in regulating the proliferation, but also correlates with the metastasis of lung cancer cells. Even though it plays a key role in the regulation of metastasis, but the mechanisms are not clearly understood.

In our study, we found that hnRNPK bound to MAP 1B-LC1, which indicated hnRNPK could regulate cytoskeleton system via binding to microtubule directly. In order to study whether hnRNPK promoted the metastasis of A549 cells depending on its binding partner MAP 1B-LC1, we detected the effect of knockdown of MAP 1B-LC1 on the metastatic capacity of lung cancer cells stably overexpressing hnRNP K. The results demonstrated that knockdown of MAP 1B-LC1 suppressed the metastasis of lung cancer cells, which suggested MAP 1B-LC1 was one regulator of microtubule cytoskeleton system and promoted the stability of microtubule system during TGF-β1-induced EMT.

E-cadherin downregulation is one of marked features during TGF-β1-induced EMT. hnRNP K plays a role in EMT of lung cancer cells depending on the translational regulation of E-cadherin mRNA 3’UTR [28]. The translation expression of mRNP is usually suppressed unless hnRNPs were phospharylated and their binding mRNAs were relieved. These need adjustment of time and space for mRNAs. The interaction of hnRNP K with MAP 1B-LC1 provides basis for these adjustment of time and space regulation. Because LC1 could binds microtubulin directly, hnRNP K may regulate the metastasis of A549 cells depending on microtubulin skeleton system. We showed that the role of MAP 1B-LC1 in regulating the migration of A549 cells induced by TGF-β1. Knockdown of MAP 1B-LC1 inhibited the EMT of A549 cells as well. In order to investigate how the interaction influences the metastasis of lung cancer cells, we enhanced hnRNPK overexpression in A549 cells, which enhanced migration of A549 cells after TGF-β1 treatment. Whereas, the improved metastatic ability was decreased by knockdown of MAP 1B-LC1. These indicated LC1 is crucial for hnRNPK to promote lung cancer cell metastasis under treatment with TGF-β1.

MAP 1B exerts effects on neural cell growth via promoting cytoskeleton assembly. MAP 1B is hydrolyzed to MAP 1B-HC and MAP 1B-LC1, which is involved in maintaining cytoskeleton stability. MAP 1B upregulation promotes α-tubulin acetylation, which leads to resistance to microtubule depolymerization [29]. These imply that hnRNPK may promote lung cancer cells migration via regulating microtubule stability. LC1 is shown to enhanced microtubule stability and alpha tubulin acetylation [29] and may be involved in Ca_v_2.2 channel functional expression by the ubiquitination/degradation pathway [30]. Acetylated ɑ-tubulin, which plays an important role in microtubule stabilization and cell morphology, could serve as a novel regulator and marker of EMT [21]. So, we inferred that hnRNPK regulate microtubule stability and the acetylation of ɑ-tubulin, and promote cell movement via MAP 1B-LC1. This inference was confirmed by our results. However, although hnRNK is involved in regulating the acetylation of ɑ-tubulin during TGF-β-induced EMT, but the regulating is not associated with MAP 1B-LC1. These results suggested that hnRNP K enhance microtubule stability via interacting with MAP 1B-LC1, but may regulate the acetylation of ɑ-tubulin through other signaling pathway.

Future development of small molecules that selectively target the interaction between MAP 1B-LC1 and hnRNP K could be useful for inhibiting the metastasis of lung cancer cells, which will accelerate the development of novel targets for lung cancer treatment. However, more detailed studies are needed to fully illustrate the roles of interaction between MAP 1B-LC1 and hnRNP K in microtubule stability. For exmaple, what modification hnRNP K undergoes to bind to MAP 1B-LC1 for regulating cytoskeleton under stimulation.

## Conclusions

**Fig. 8:**
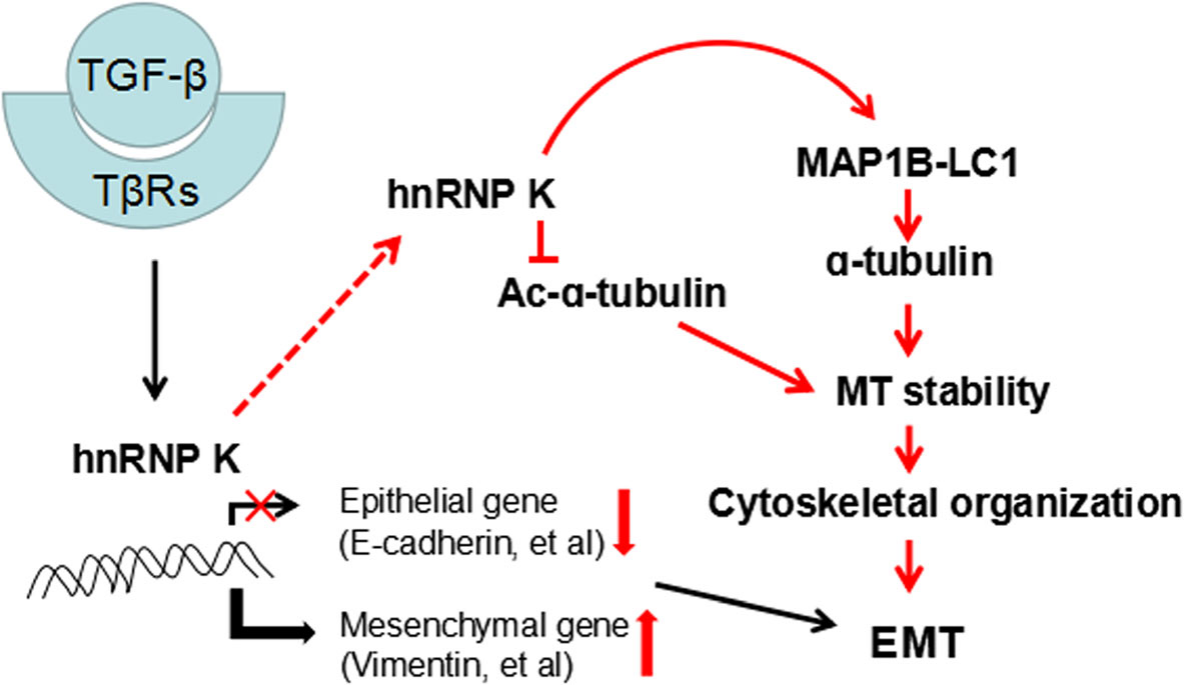
Working modeling for the molecular mechanism underlying the involvement of hnRNP K during TGF-β-induced EMT

In this study, the results demonstrated hnRNPK promoted metastasis and microtubule stability of lung cancer cells via interaction with LC1 after TGF-β1 treatment, indicating that MAP 1B-LC1 is required for hnRNP K-mediated EMT induced by TGF-β1 (Fig.8). The interaction of hnRNP K and MAP 1B-LC1 is important in regulating TGF-β1-induced EMT. These findings improve our understanding on the molecular mechanism underlying the involvement of hnRNP K during TGF-β-induced EMT, which may identify new therapeutic strategies for patients with NSCLC.

## Additional file

Additional file 1: **Table S1.** hnRNP K interacting proteins identified in this study (DOCX 17 kb)

## Data Availability

The datasets used and/or analyzed during the current study are available from the corresponding author on reasonable request.

## Abbreviations

Co-IP: Co-immunoprecipitation
EMT: Epithelial-to-mesenchymal transition
hnRNP K: Heterogeneous nuclear ribonucleoprotein K
IPTG: Isopropyl β-D-thiogalactopyranoside
MAP 1B-LC1: Microtubule-associated protein 1B light chain
NSCLC: Non-small-cell lung cancer
PVDF: Polyvinylidene-difluoride
