# Supplementary material for: Interaction of hnRNP K with MAP 1B-LC1 promotes TGF-β1-mediated epithelial to mesenchymal transition in lung cancer cells
Source: BMC Cancer. 2019 Sep 6;19:894. doi: 10.1186/s12885-019-6119-x (PMC6731588; doi:10.1186/s12885-019-6119-x)
Supplement: Supplementary file 1 — Table S1. hnRNP K interacting proteins identified in this study (DOCX 17 kb) [file 12885_2019_6119_MOESM1_ESM.docx]

**Additional file 1: Table S1 hnRNP K interacting proteins identified in this study**

| Protein IDs | Protein names | Gene names |
| --- | --- | --- |
| IPI00005792 | Polyadenylate-binding protein 2 | PABPN1 |
| IPI00007208 | ATP-dependent RNA helicase DDX41 | DDX41 |
| IPI00017617 | ATP-dependent RNA helicase DDX5 | DDX5 |
| IPI00023785 | ATP-dependent RNA helicase DDX17 | DDX17 |
| IPI00902597 | DNA topoisomerase 1 | TOP1 |
| IPI00008557 | Insulin-like growth factor 2 mRNA-binding protein 1 | IGF2BP1;CRDBP;  VICKZ1;ZBP1 |
| IPI00179713 | Insulin-like growth factor 2 mRNA-binding protein 2 | IGF2BP2 |
| IPI00658000 | Insulin-like growth factor 2 mRNA-binding protein 3 | IGF2BP3 |
| IPI00008868 | Microtubule-associated protein 1B;MAP1 light chain LC1 | MAP1B-LC1 |
| IPI00019502 | Myosin-9;Myosin heavy chain 9 | MYH9 |
| IPI0078955 | Putative uncharacterized protein MATR3 | MATR3;KIAA0723 |
| IPI00182757 | Protein KIAA1967 | KIAA1967 |
| IPI00456887 | Heterogeneous nuclear ribonucleoprotein U-like protein 2 | HNRNPUL2 |
| IPI00027834 | Heterogeneous nuclear ribonucleoprotein L | HNRNPL |
| IPI00472054 | Constitutive coactivator of PPAR-gamma-like protein 1 | FAM120A |
| IPI00297982 | Eukaryotic translation initiation factor 2 subunit 3 | EIF2S3 |
| IPI00872430 | 40S ribosomal protein S8 | RPS8 |
| IPI00217465 | Histone H1.2; H1c (HIST1H1C), mRNA | HIST1H1C |
| IPI00217466 | Histone H1.2; H1c (HIST1H1D), mRNA | HIST1H1D |
| IPI00217467 | Histone H1.2; H1c (HIST1H1E), mRNA | HIST1H1E |
| IPI00183626 | Polypyrimidine tract binding protein 1 | PTBP1; HNRNPI |
| IPI00021439 | Beta-actin | ACTB |
